# FCGBP Is a Promising Prognostic Biomarker and Correlates with Immunotherapy Efficacy in Oral Squamous Cell Carcinoma

**DOI:** 10.1155/2022/8443392

**Published:** 2022-06-12

**Authors:** Ting Shen, Jianying Zhang, Yali Wang, Binjie Liu

**Affiliations:** Xiangya Stomatological Hospital & Xiangya School of Stomatology, Central South University & Hunan key Laboratory of Oral Health Research & Hunan 3D Printing Engineering Research Center of Oral Care & Hunan Clinical Research Center of Oral Major Diseases and Oral Health, Changsha, 410008 Hunan, China

## Abstract

Oral squamous cell carcinoma (OSCC) is one of the most common malignancies of the head and neck. In OSCC patients, the prognosis was dramatically different. In this research, we aimed to study the expressions and prognostic values of IgG Fc binding protein (FCGBP) in OSCC patients. The expression of FCGBP was analyzed using TCGA datasets and GEO datasets. FCGBP was evaluated for its predictive significance in OSCC patients by the use of a Kaplan-Meier and Cox regression model. Enrichment analysis for the GO and KEGG databases were conducted. CIBERSORT used TCGA datasets to show immune cell infiltration. In addition, researchers looked into the relationships between FCGBP and immune cells. The levels of FCGBP in OSCC cells was examined through the use of RT-PCR. FCGBP overexpression was tested for its effects on OSCC cell proliferation and invasion using CCK-8 and Transwell assays. We observed that FCGBP expressions were distinctly downregulated in OSCC specimens compared with nontumor tissues in both TCGA and GEO datasets, which was further confirmed by RT-PCR. OSCC patients with advanced clinical stages and poor prognoses had lower levels of FCGBP expression. Many immune-related biological activities and signaling pathways were found to be considerably abundant in KEGG tests and GO analysis results. The correlation analysis indicated that FCGBP was associated with a number of immune cells in a positive way. We found that FCGBP expressions were strongly and distinctly linked to the expressions of known immunological checkpoints, and FCGBP expression had significant positive connections with tumor mutational burden. FCGBP upregulation distinctly slowed the growth and invasion of OSCC cells in functional experiments. FCGBP has the potential to be a therapeutic target for OSCC and a biomarker for OSCC patients' prognosis.

## 1. Introduction

The sixth most prevalent malignancy in the world is head and neck cancer [1]. Oral squamous cell carcinoma (OSCC) is the most common subtype of head and neck cancer [2]. In 2018, more than 300,000 new instances of OSCC were discovered, and 170,000 people died as a result of the disease [3, 4]. While advances in the treatment of OSCC with surgery, radiation, and chemotherapy have been made recently, the disease's invasive, metastatic, and recurring characteristics still restrict the treatment's efficacy, posing a major threat to the health of patients [5, 6]. In the five years following diagnosis, the survival rate for people with OSCC is still only 50-60% [7]. OSCC is thought to be mostly caused by smoking, but other risk factors include excessive alcohol use, betel nut chewing, and infection with the human papillomavirus [8]. Thus, sensitive markers are desperately needed to aid in the diagnosis of OSCC, predict clinical outcomes, and serve as a basis for customized therapy.

IgG Fc binding protein (FCGBP) was first discovered as an Fc portion of the IgG molecule binding site in intestinal and colonic epithelia [9]. In ulcerative colitis, a chronic inflammatory illness that increases the risk of colon cancer, FCGBP has been found to be downregulated [10]. In addition, gallbladder cancer has low levels of FCGBP expression, and this protein is a critical regulator of the TGF-1-induced EMT progress [11]. Meanwhile, prostate cancer patients with low levels of this gene's expression have been found to have a faster course of disease [12]. Although its molecular role is still unclear, various studies have revealed that it may be connected to the body's innate immunity [13, 14]. There was a lack of understanding of the expression and functions of FCGBP in OSCC. In this research, we set out to examine the expressions and prognostic values of FCGBP in OSCC, as well as the link between FCGBP and immune cells and immune regulation-related genes.

## 2. Materials and Methods

### 2.1. Cell Culture and Reagents

Normal oral mucosal HOMEC cells and OSCC cells (TSCCA, SCC15, and CAL27) were purchased from American Type Tissue Collection (ATCC, Massachusetts, USA). DMEM (Gibco, Shanghai, China) with 10% FBS (GIBCO, Guangzhou, China) and 1% penicillin-streptomycin (PriCells, Wuhan, China) was used to cultivate the cells in a 37°C incubator with 5 percent CO_2_.

### 2.2. Cell Transfection

The overexpressing plasmids, including pcDNA3.1 and pcDNA3.1-FCGBP, were purchased from B&H Biology (Guangzhou, Guangdong, China). Lipofectamine 2000 reagent kits (Invitrogen, Jinsui, Shanghai, China) were used for cell transfection in line with the kit instructions.

### 2.3. qRT-PCR Analysis

Following the manufacturer's instructions, TRIzol (Invitrogen, USA) was applied to collect total RNAs from OSCC cells. HiScript cDNA Synthesis Kit (Vazyme, China) was applied to reverse-transcribe the common genes using a gDNA wiper. Using the ChamQ Universal SYBR qPCR Master Mix, the target genes were measured using qPCR amplification (Vazyme). GAPDH was used as the internal control. Gene amplification levels were measured using the 2^−*ΔΔ*Ct^ methods. All the PCR primer sequences used in this study were included in Table S1.

### 2.4. Cell Counting Kit-8 (CCK-8) Assay

At a density of 1 × 10^4^/mL, OSCC cell suspensions were injected into a 96-well plate. CCK-8 solution (10 L) was added to each well every day for three days at the same time (Vazyme, Nanjing, Jiangsu, China). After a two-hour incubation, we used a microplate reader (Thermo-Fisher Scientific) to detect the absorbance at 450 nm.

### 2.5. Cell Invasion

Upper Transwell chambers were filled with the transfected SCC15 and CAL27 cells, which had been cultured in 200 l of serum-free RPMI-1640 media (Corning Inc.; 24-well insert, pore size 8 mm). Matrigel (BD Biosciences, Haidian, Beijing, China) was applied to the Transwell membrane prior to use. It was after 24 hours of incubation at 37°C that the cells were fixed and then stained for 15 minutes at room temperature with 0.5% crystal violet solution. A light microscope was used to count the invading cells. Analyzing the photos required the use of ImageJ software. A total of five random fields were used to take images.

### 2.6. Data Preparation

Gene expression profiling and clinical data from The Cancer Genome Atlas (TCGA) database were downloaded using the GDC Data Transfer Tool, which includes RNA-Seq of Transcriptome Profiling and Clinical data for OSCC primitive data (gingiva, the anterior 2/3 of the tongue, buccal mucosa, palate, floor of mouth, oral cavity, and so on). After that, six samples were discarded due to the poor quality of the clinical data they included. As a result, we gathered 278 OSCC and 31 nontumor samples for further assays. OSCC' gene expression profiles were downloaded from GEO datasets: GSE30784. The platform of GSE30784 was based on GPL570 (Affymetrix Human Genome U133 Plus 2.0 Array). GSE30784 contained 167 OSCC and 45 normal oral tissue samples.

### 2.7. Differentially Expressed Gene Analysis

According to the median expressions of FCGBP, the expression data (HTseq-Counts) were separated into low and high expressing groups before being subjected to unpaired Student's *t*-test within the DESeq2 R program for further examination. ∣log2‐fold change (FC) | >1 and adjusted *p* < 0.05 were considered thresholds for the DEGs.

### 2.8. GO and KEGG Enrichment Assays

The clusterProfiler was implemented in R, an open-source programming environment, and was released under Artistic License 2.0 within Bioconductor project [15]. clusterProfiler R software was applied to carry out KEGG pathway assays and Gene Ontology (GO) analysis of FCGBP and its interacting proteins with an FDR cutoff of 0.05 [16]. The “ggplot2” package's histogram was used to display the findings.

### 2.9. Immune Infiltration Analysis

CIBERSORT (https://cibersortx.stanford.edu) was applied to evaluate the proportion of 22 immune infiltrating cell types in each sample of the TCGA cohort. After removing samples with a *p* value of < 0.05, an empirical *p* value for deconvolution of each case was determined. As a result, we looked for 22 distinct subtypes of immune invading cells that were linked to OSCCs and FCGBP expression. The purity-corrected partial Spearman's correlation coefficient was utilized to examine the connection between FCGBP expression and immune infiltration.

### 2.10. Correlation Analysis

Correlations between FCGBP and other immunological checkpoints (such as BTLA, TMIGD2, ADORA2A, and CD200R1) were examined using Pearson's correlation coefficient. The data were exhibited as heat maps by the use of “pheatmap” package.

### 2.11. Calculation of TMB in OSCC Patients

In the evaluated coding regions of the genome, TMB was defined as the number of insertion/deletion (indel) and replacement mutations per megabase. Cases with silent mutations, 3 or 5 untranslated sections, or tiny in-frame insertions or deletions that did not result in a change in amino acid sequence were all eliminated. We calculated the TMB scores by dividing the total number of somatic mutations through the size of the exome.

### 2.12. Statistical Analysis

Unpaired *t*-tests and one-way analysis of variance were employed to compare continuous variables in this study. Correlation and Wilcoxon rank-sum tests were used to compare the infiltration of immunocytes between the high- and low-expression groups of FCGBP. Kaplan-Meier survival analysis, log-rank test, and Cox regression model were used to explore the prognostic value of FCGBP expression in OSCC. Statistical significance is defined as a *p* value of less than 0.05. We used R (v.3.6.1, R Core Team, Boston, MA, USA) and SPSS 26.0 (IBM Corporation, Illinois, USA) for the above data analysis.

## 3. Results

### 3.1. The Distinct Upregulation of FCGBP Expression in OSCC and Its Association with Clinical Factors

**Figure 1:**
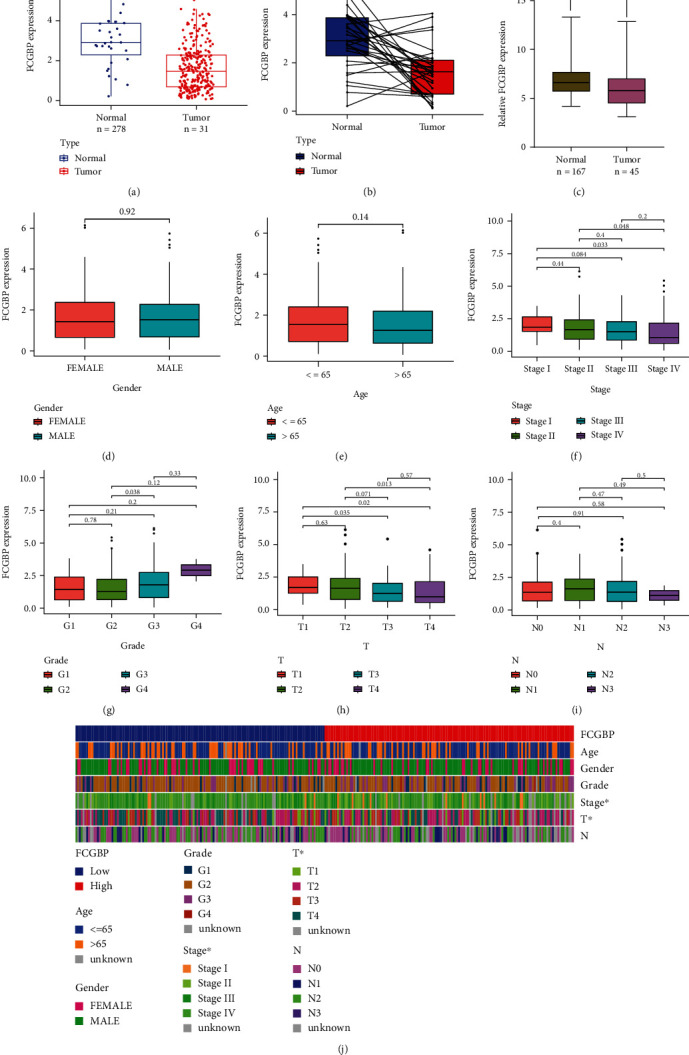
The expressing pattern of FCGBP in OSCC patients. (a, b) The expression of FCGBP was distinctly decreased in OSCC samples compared with nontumor samples from TCGA datasets. (c) Lower levels of FCGBP were observed in OSCC in OSCC samples than nontumor samples from GSE30784 datasets. (d–i) The association between FCGBP expression and gender, age, stage, grade, T stage, and N stages. (j) Heat map showed the associations between FCGBP expressions and clinical factors. ^∗^*p* < 0.05, ^∗∗∗^*p* < 0.001.

We analyzed TCGA datasets and observed that FCGBP expressions were distinctly decreased in OSCC samples compared with nontumor samples (Figures 1(a) and 1(b)). Then, we further confirmed this result using GSE30784 (Figure 1(c)). Our findings suggested FCGBP as a possible regulator in OSCC progression. Then, we further analyzed the association of FCGBP with clinical factors. However, the expression of FCGBP was not associated with the gender and age of OSCC patients (Figures 1(d) and 1(e)). Interesting, we observed that OSCC specimens with advanced stages showed a lower level than those with early stages (Figures 1(f)–1(i)). Moreover, the heat map showed the associations between FCGBP expressions and several clinical factors (Figure 1(j)).

### 3.2. The Prognostic Values of FCGBP Expression in OSCC

**Figure 2:**
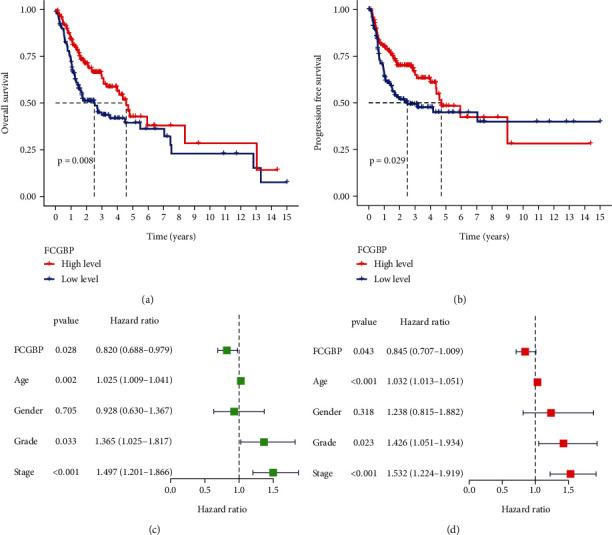
The prognostic value of FCGBP in OSCC patients. (a, b) Kaplan-Meier curves of the overall survival and progression-free survival of OSCC patients. (c, d) Univariate and multivariate assays of overall survival in OSCC cases.

For this study, we compared the overall survival rates of patients with high and low FCGBP expressions to see if there was a correlation between the two groups. The results showed that patient's overall survival and progression-free survival time were shown to be considerably longer in patients with high FCGBP expression compared to patients with low FCGBP expression (Figures 2(a) and 2(b)). For the purpose of determining if FCGBP expression was an independent prognostic factor in OSCC patients, we conducted univariate and multivariate analyses. OSCC patients' overall survival was found to be substantially linked with their grade, stage, and expression of FCGBP in the univariate analysis (Figure 2(c)). In addition, multivariate assays confirmed that FCGBP expression was an independent predictive factor for overall survival (HR = 0.845; 95% CI, 0.707-1.009; *p* = 0.043) in patients with OSCC (Figure 2(d)). These data revealed that FCGBP expression may a promising biomarker for OSCC patients.

### 3.3. Coexpression Genes of FCGBP in OSCC Samples

**Figure 3:**
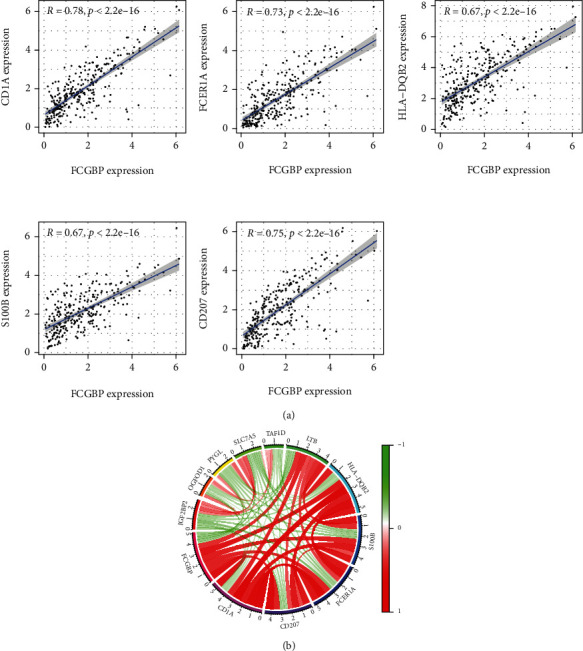
The coexpression analysis of FCGBP in OSCC samples. (a) FCGBP expression was positively associated with CD1A, CD207, FCER1A, HLA-DQB2, and S100B. (b) Circle graph was established.

Then, we also analyzed the coexpression genes of FCGBP in OSCC samples. As exhibited in Figure 3(a), our group observed that FCGBP expression was positively related to CD1A, CD207, FCER1A, HLA-DQB2, and S100B. Moreover, circle graph was established (Figure 3(b)).

### 3.4. GO and KEGG Assays

**Figure 4:**
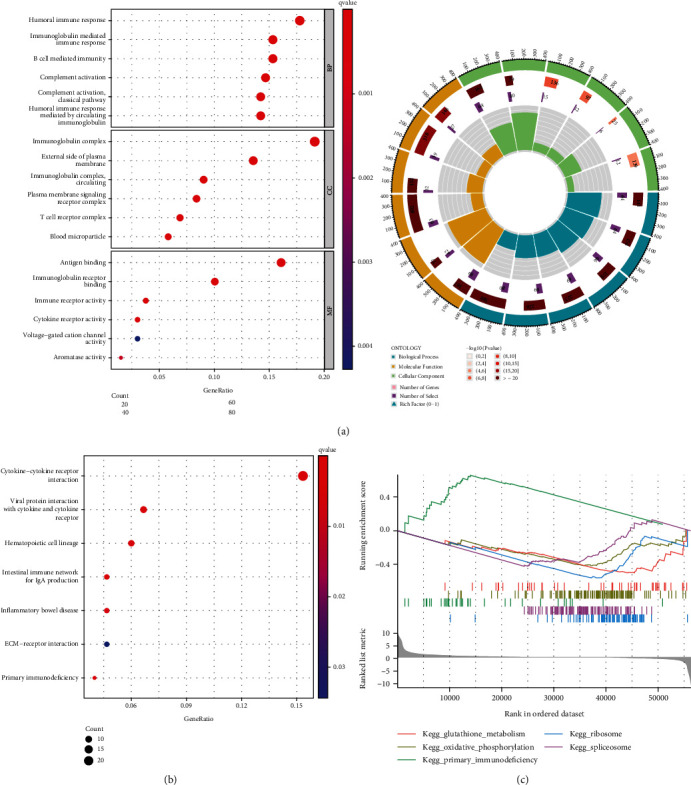
Functional analysis based on the DEGs between the two groups in the TCGA cohort. (a) Bubble graph for GO enrichment. (b) Bar plot graph for KEGG pathways. (c) Enrichment plots from GSEA.

FDR < 0.05 and ∣log2FC  | >1 were used to identify the DEGs using the “limma” R package. 675 DEGs were discovered between the TCGA cohorts with high and low FCGBP expressions. Heat map showed the top 50 downregulated or upregulated genes (Figure S1). The biological roles of FCGBP in OSCC were elucidated using GO enrichment and KEGG pathway analyses. Many immune-related molecular functions were shown to be enriched by GO analysis (Figure 4(a)). Likewise, KEGG assays indicated that DEGs were distinctly enriched in cytokine-cytokine receptor interaction, hematopoietic cell lineage, intestinal immune network for IgA production, inflammatory bowel disease, ECM-receptor interaction, and primary immunodeficiency (Figures 4(b) and 4(c)).

### 3.5. Correlation of FCGBP with the Proportion of Tumor-Infiltrating Immune Cells (TICs)

**Figure 5:**
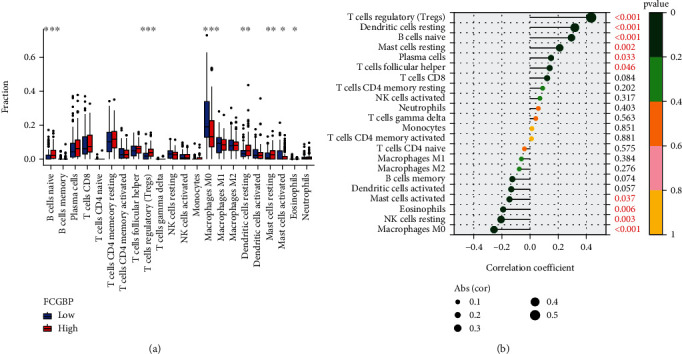
Correlations of FCGBP expressions with immune infiltration levels in the OSCC samples. (a) Assays of differential immune cells between the low and high FCGBP expression groups in TCGA. (b) Correlation between FCGBP and infiltrating immune cells in OSCC. ^∗^*p* < 0.05, ^∗∗^*p* < 0.01, and ^∗∗∗^*p* < 0.001.

CIBERSORT algorithm was used to analyze the fraction of tumor-infiltrating immune subsets, and 21 different types of immune cell profiles in OSCC patients were completed to further validate the association between FCGBP expressions and the immune microenvironment. As shown in Figure 5(a), we found that B cells naïve, T cells regulatory (Tregs), macrophages M0, dendritic cell resting, mast cells resting, mast cells activated, and eosinophils exhibited a dysregulated level between high-FCGBP-expressions groups and low-FCGBP-expressions groups. In addition, correlation analysis revealed that FCGBP was positively correlated with T cells regulatory (Tregs), dendritic cell resting, B cell naïve, mast cell resting, plasma cells, and T cell follicular helper. However, FCGBP was negatively correlated with mast cells activated, eosinophils, NK cell resting, and macrophage M0 (Figure 5(b) and Figure S2).

### 3.6. Relationship between Immune Checkpoints and FCGBP

**Figure 6:**
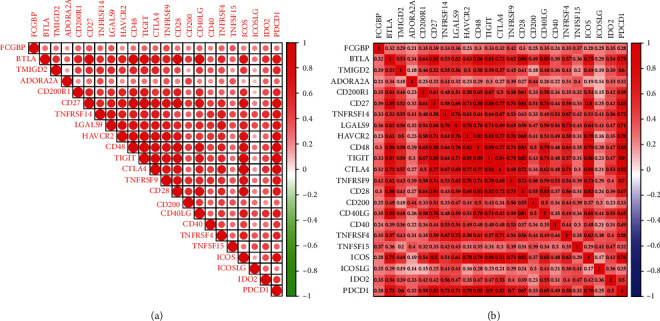
(a, b) The correlations between FCGBP and immune checkpoints in OSCC samples.

Figures 6(a) and 6(b) showed the connections between immunological checkpoints and FCGBP. We observed that the distinct associations existed between the expressions of FCGBP and the expressions of several immune checkpoints including BTLA, TMIGD2, ADORA2A, CD200R1, CD27, TNFRSF14, LGALS9, HAVCR2, CD48, TIGIT, CTLA4, TNFRSF9, CD28, CD200, CD40LG, CD40, TNFRSF4, TNFSF15, ICOS, ICOSLG, IDO2, and PDCD1. Our findings revealed a potential synergy of FCGBP with immune checkpoints.

### 3.7. FCGBP Expressions Were Associated with Tumor Mutational Burden (TMB)

**Figure 7:**
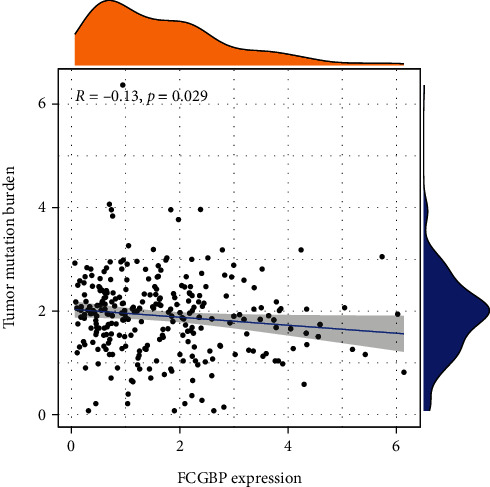
The correlations between FCGBP expression and TMB in OSCC samples.

FCGBP's role in the immunological mechanism and immune response of the tumor microenvironment (TME) was examined via studying the correlations between FCGBP expressions and TMB. TMB in the tumor microenvironment is linked to anti-tumor immunity and could predict the efficacy of tumor immunotherapy. FCGBP expression was found to have a positive correlation with TMB in OSCC cases (Figure 7).

### 3.8. The Expression and Function of FCGBP in OSCC

**Figure 8:**
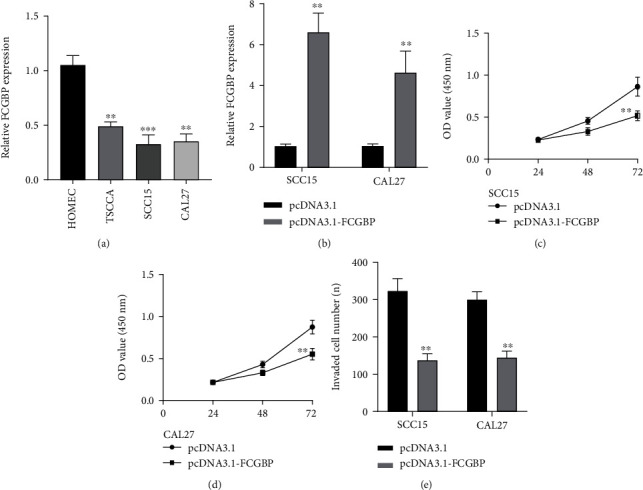
The oncogenic roles of FCGBP in OSCC progression. (a) RT-PCR for the expression of FCGBP in three OSSC cell lines (TSCCA, SCC15, and CAL27) sand HMOEC cells. (b) FCGBP-overexpressed SCC15 and CAL27 cells were established, which was confirmed by RT-PCR. (c, d) CCK-8 assay was applied to detect the effects of FCGBP upregulation on the proliferation of SCC15 and CAL27 cells. (e) Transwell invasion assays. ^∗∗^*p* < 0.01, ^∗∗∗^*p* < 0.001.

To explore whether FCGBP was dysregulated in OSCC, we performed RT-PCR to examine the expression of FCGBP in OSCC cells, finding that FCGBP expression was distinctly decreased in TSCCA, SCC15, and CAL27 cells (Figure 8(a)). Then, we used pcDNA3 and 1-FCGBP to increase the expression of FCGBP in SCC15 and CAL27 cells, which was demonstrated by the use of RT-PCR (Figure 8(b)). CCK-8 analysis was used to examine the functions of FCGBP overexpression on the proliferation of OSCC cells, and we observed that FCGBP overexpression distinctly suppressed the proliferation of SCC15 and CAL27 cells (Figures 8(c) and 8(d)). Besides, the results of Transwell experiments revealed that overexpression of FCGBP distinctly suppressed the invasion of SCC15 and CAL27 cells (Figure 8(e)).

## 4. Discussion

Screening new markers for OSCC may improve treatment options and predict the outcomes of patients [17, 18]. Recently, growing studies have indicated that some functional genes can serve as diagnostic or prognostic markers in many tumors, including OSCC [19, 20]. Furthermore, tumor-related genes such as METTL3 and METTL3 have been identified as independent prognostic factors for OSCC [21, 22]. However, the clinical importance of several genes in OSCC has yet to be established.

Based on our data, we identified a novel OSCC-related gene, FCGBP which showed a low level in OSCC specimens compared with nontumor specimens. Previously, many studies have indicated that the dysregulation of FCGBP was involved in the progression of several tumors. For instance, ovarian cancer patients with high levels of FCGBP had a lower overall survival and disease-specific survival than those with lower levels of the protein [23]. In addition, as a tumor promotor in ovarian cancer, FCGBP may contribute to the polarization of macrophages into M2 cells. Yuan et al. reported that when it came to primary and metastatic liver cancer, CGBP RNA expression was significantly reduced. Both overall survival and disease-free survival were negatively impacted by FCGBP positive in the liver metastatic population [24]. However, the potential power of FCGBP in OSCC has not been determined. We further provided evidences that high FCGBP expression was associated with favorable prognosis, which was consistent with its effects in ovarian cancer, colorectal cancer, and gallbladder cancer. More importantly, we confirmed FCGBP as an independent prognostic indicator for overall survival in patients with OSCC. In addition, we also analyzed the potential function of FCGBP overexpression on the progression of OSCC and observed that FCGBP upregulation distinctly suppressed the proliferation and invasion of OSCC cells, indicating it as an oncogene in OSCC.

For the exploration of the potential functions of FCGBP in OSCC progression, we performed GO and KEGG assays using the dysregulated genes between OSCC samples with high FCGBP expression and OSCC samples with low CGBP expression. Numerous immune-related molecular activities were found to be considerably enhanced by GO analysis. KEGG analyses still found that genes were significantly enriched in cytokine-cytokine receptor interaction, viral protein interaction with cytokine and cytokine receptor, hematopoietic cell lineage, intestinal immune network for IgA production, inflammatory bowel disease, ECM-receptor interaction, and primary immunodeficiency. Our findings suggested FCGBP may be involved in immunosuppressive status of OSCC.

Tumor growth, progression, and metastasis are all influenced by the interplay between immune cells and tumor cells in the tumor microenvironment [25]. As one of the most prominent immune suppressive cells in the TME, tumor-associated macrophages (TAMs) play a key role in promoting tumor growth through controlling the TME [26]. Angiogenesis, immunosuppression, and precancerous metastasis are all supported by TAMs, which are critical in the promotion of tumor progression [27, 28]. They also play a role in tumor genesis and progression. In this study, we found that FCGBP was positively correlated with T cell regulatory (Tregs), dendritic cells resting, B cell naïve, mast cell resting, plasma cells, and T cell follicular helper. However, FCGBP was negatively correlated with mast cell activated, eosinophils, NK cell resting, and macrophages M0. There is a putative mechanism by which FCGBP affects OSCC survival by increasing the number of T and B cells, which is consistent with FCGBP's beneficial effects on OSCC survival through increased T and B cell numbers [29, 30]. Cancer immunotherapy relies heavily on immunological checkpoints, which are a collection of chemicals that can stimulate or suppress the immune system [31, 32]. Immune checkpoints such as IDO1, PD-L2, PD-1, and LAG3 were downregulated in OSCC relative to healthy specimens [33, 34]. We observed that distinct relationships existed between CD96 expressions and expressions of immune checkpoints. This suggested a potential synergy of FCGBP with known immune checkpoints. Moreover, a number of studies have found that TMB can predict the efficacy of immunotherapy as well as the response rate to immunotherapy in different cancers, including OSCC. We also confirmed that FCGBP expression had significant positive associations with TMB in OSCC. A high TMB score indicates a poor prognosis for OSCC patients; thus, immunotherapy could be beneficial for those with mutated genes.

Nevertheless, our study has several limitations. Firstly, this was a retrospective study, so there may be biases in the selection of variables, resulting in a loss of data accuracy. Secondly, data used in this work was obtained from public sources, and further experiments in vivo and in vitro are required to confirm the mechanism by which FCGBP affected the occurrence and progression of OSCC.

## 5. Conclusion

Overall, our findings revealed that the expressions of FCGBP were elevated in OSCC specimens, and it could be a new marker for the prediction of outcomes of OSCC patients. Besides, high FCGBP expressions were associated with immunosuppressive tumor microenvironment characteristics. Our findings may facilitate the identification of novel biomarkers for evaluating tumor stage, aiding drug development, and improving treatment efficiency.

## Data Availability

The data used to support the findings of this study are available from the corresponding author upon request.

## Supplementary Materials

Supplementary 1 Figure S1: heat map showed the top 50 downregulated or upregulated genes between high-FCGBP-expressions groups and low-FCGBP-expressions groups. Figure S2: correlation analysis between FCGBP expression and 22 immune cells.

Supplementary 2 Table S1: the primer sequences included in this study.

## References

[1] Chow L. Q. M. (2020). Head and neck cancer. *The New England Journal of Medicine*.

[2] Yokota T., Homma A., Kiyota N. (2020). Immunotherapy for squamous cell carcinoma of the head and neck. *Japanese Journal of Clinical Oncology*.

[3] Almangush A., Mäkitie A. A., Triantafyllou A. (2020). Staging and grading of oral squamous cell carcinoma: an update. *Oral Oncology*.

[4] Sasahira T., Kirita T. (2018). Hallmarks of cancer-related newly prognostic factors of oral squamous cell carcinoma. *International Journal of Molecular Sciences*.

[5] Alves A. M., Diel L. F., Lamers M. L. (2018). Macrophages and prognosis of oral squamous cell carcinoma: a systematic review. *Journal of Oral Pathology & Medicine*.

[6] Leemans C. R., Snijders P. J. F., Brakenhoff R. H. (2018). The molecular landscape of head and neck cancer. *Nature Reviews. Cancer*.

[7] Brands M. T., Brennan P. A., Verbeek A. L. M., Merkx M. A. W., Geurts S. M. E. (2018). Follow-up after curative treatment for oral squamous cell carcinoma. A critical appraisal of the guidelines and a review of the literature. *European Journal of Surgical Oncology*.

[8] Paderno A., Morello R., Piazza C. (2018). Tongue carcinoma in young adults: a review of the literature. *Acta Otorhinolaryngologica Italica*.

[9] Hoffmann W. (2021). Trefoil factor family (TFF) peptides and their different roles in the mucosal innate immune defense and more: an update. *Current Medicinal Chemistry*.

[10] Yang W., Shi J., Zhou Y. (2019). Integrating proteomics and transcriptomics for the identification of potential targets in early colorectal cancer. *International Journal of Oncology*.

[11] Xiong L., Wen Y., Miao X., Yang Z. (2014). NT5E and FcGBP as key regulators of TGF-1-induced epithelial-mesenchymal transition (EMT) are associated with tumor progression and survival of patients with gallbladder cancer. *Cell and Tissue Research*.

[12] Gazi M. H., He M., Cheville J. C., Young C. Y. (2008). Downregulation of IgG Fc binding protein (Fc gammaBP) in prostate cancer. *Cancer Biology & Therapy*.

[13] Yan T., Tian D., Chen J. (2021). FCGBP is a prognostic biomarker and associated with immune infiltration in glioma. *Frontiers in Oncology*.

[14] Houben T., Harder S., Schlüter H., Kalbacher H., Hoffmann W. (2019). Different forms of TFF3 in the human saliva: heterodimerization with IgG fc binding protein (FCGBP). *International Journal of Molecular Sciences*.

[15] Gentleman R. C., Carey V. J., Bates D. M. (2004). Bioconductor: open software development for computational biology and bioinformatics. *Genome Biology*.

[16] Yu G., Wang L. G., Han Y., He Q. Y. (2012). clusterProfiler: an R package for comparing biological themes among gene clusters. *OMICS*.

[17] Huang G. Z., Wu Q. Q., Zheng Z. N. (2020). M6A-related bioinformatics analysis reveals that HNRNPC facilitates progression of OSCC via EMT. *Aging (Albany NY)*.

[18] Yang Z., Liang X., Fu Y. (2019). Identification of AUNIP as a candidate diagnostic and prognostic biomarker for oral squamous cell carcinoma. *eBioMedicine*.

[19] Ueda S., Goto M., Hashimoto K. (2021). Salivary *CCL20* level as a biomarker for oral squamous cell carcinoma. *Cancer Genomics Proteomics*.

[20] Ueda S., Goto M., Hashimoto K. (2021). Salivary *CPLANE1* levels as a biomarker of oral squamous cell carcinoma. *Anticancer Research*.

[21] Zhao W., Cui Y., Liu L. (2020). METTL3 facilitates oral squamous cell carcinoma tumorigenesis by enhancing c-Myc stability via YTHDF1-mediated m^6^A modification. *Molecular Therapy-Nucleic Acids*.

[22] Mao L., Zhuang R., Qin L. (2020). CCL18 overexpression predicts a worse prognosis in oral squamous cell carcinoma (OSCC). *Neoplasma*.

[23] Wang K., Guan C., Shang X. (2021). A bioinformatic analysis: the overexpression and clinical significance of FCGBP in ovarian cancer. *Aging (Albany NY)*.

[24] Yuan Z., Zhao Z., Hu H. (2021). IgG fc binding protein (*FCGBP*) is down-regulated in metastatic lesions and predicts survival in metastatic colorectal cancer patients. *Oncotargets and Therapy*.

[25] Arneth B. (2020). Tumor microenvironment. *Medicina (Kaunas, Lithuania)*.

[26] Pan Y., Yu Y., Wang X., Zhang T. (2020). Tumor-associated macrophages in tumor immunity. *Frontiers in Immunology*.

[27] Cassetta L., Pollard J. W. (2020). Tumor-associated macrophages. *Current Biology*.

[28] Chen D., Zhang X., Li Z., Zhu B. (2021). Metabolic regulatory crosstalk between tumor microenvironment and tumor-associated macrophages. *Theranostics*.

[29] Cui C., Merritt R., Fu L., Pan Z. (2017). Targeting calcium signaling in cancer therapy. *Acta Pharmaceutica Sinica B*.

[30] Quan H., Fang L., Pan H. (2016). An adaptive immune response driven by mature, antigen-experienced T and B cells within the microenvironment of oral squamous cell carcinoma. *International Journal of Cancer*.

[31] Li B., Chan H. L., Chen P. (2019). Immune checkpoint inhibitors: basics and challenges. *Current Medicinal Chemistry*.

[32] Postow M. A., Sidlow R., Hellmann M. D. (2018). Immune-related adverse events associated with immune checkpoint blockade. *The New England Journal of Medicine*.

[33] Chai A. W. Y., Lim K. P., Cheong S. C. (2020). Translational genomics and recent advances in oral squamous cell carcinoma. *Seminars in Cancer Biology*.

[34] Kujan O., van Schaijik B., Farah C. S. (2020). Immune checkpoint inhibitors in oral cavity squamous cell carcinoma and oral potentially malignant disorders: a systematic review. *Cancers (Basel)*.

