# Supplementary figures and images for: FCGBP Is a Promising Prognostic Biomarker and Correlates with Immunotherapy Efficacy in Oral Squamous Cell Carcinoma
Source: J Immunol Res. 2022 Jun 12;2022:8443392. doi: 10.1155/2022/8443392 (PMC9207623; doi:10.1155/2022/8443392)

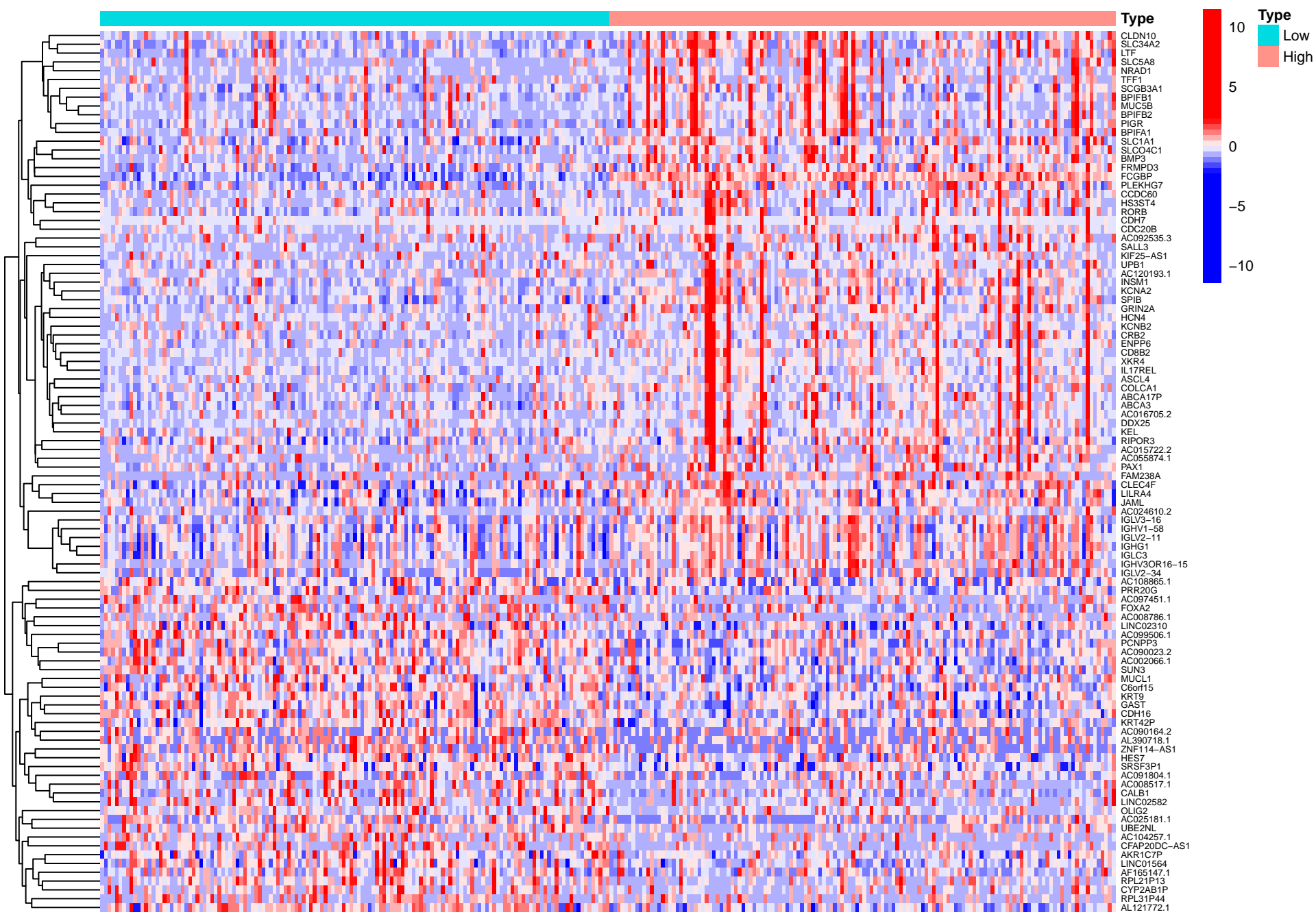

Supplement: Supplementary 1 — Figure S1: heat map showed the top 50 downregulated or upregulated genes between high-FCGBP-expressions groups and low-FCGBP-expressions groups. Figure S2: correlation analysis between FCGBP expression and 22 immune cells. [file 8443392.f1.zip › Figure S1.pdf]

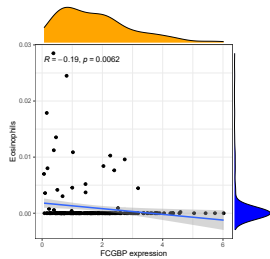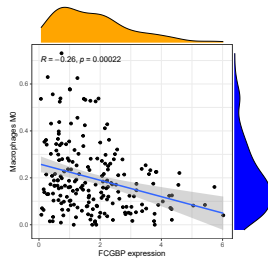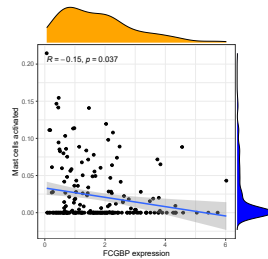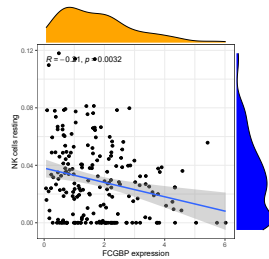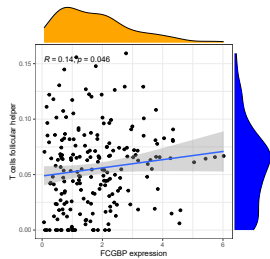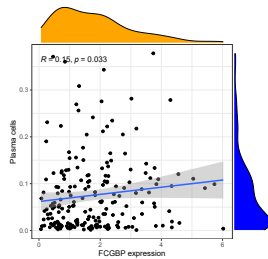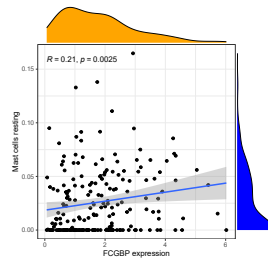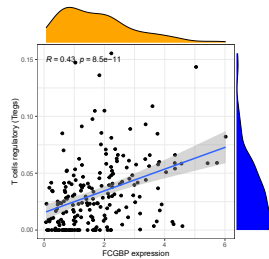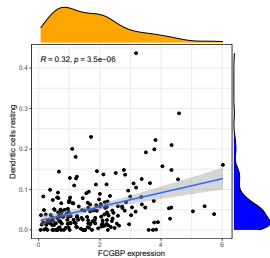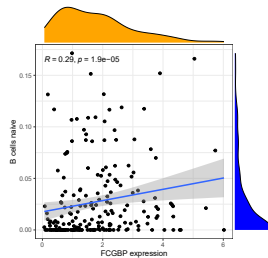

Supplement: Supplementary 1 — Figure S1: heat map showed the top 50 downregulated or upregulated genes between high-FCGBP-expressions groups and low-FCGBP-expressions groups. Figure S2: correlation analysis between FCGBP expression and 22 immune cells. [file 8443392.f1.zip › Figure S2.pdf]
