# Supplementary material for: FCGBP Is a Promising Prognostic Biomarker and Correlates with Immunotherapy Efficacy in Oral Squamous Cell Carcinoma
Source: J Immunol Res. 2022 Jun 12;2022:8443392. doi: 10.1155/2022/8443392 (PMC9207623; doi:10.1155/2022/8443392)
Supplement: Supplementary 2 — Table S1: the primer sequences included in this study. [file 8443392.f2.docx]

**Table S1** The primer sequences included in this study.

| Name | primer sequences (5’–3’) |
| --- | --- |
| FCGBP: forward | GCCAAGGCTGAGATGATAGGC |
| FCGBP: reverse | CCTGCACAGAGATGGCATAGT |
| GAPDH: forward | GGAGCGAGATCCCTCCAAAAT |
| GAPDH: reverse | GGCTGTTGTCATACTTCTCATGG |
